# Plasm YKL-40 Levels Are Associated with Hypertension in Patients with Obstructive Sleep Apnea

**DOI:** 10.1155/2019/5193597

**Published:** 2019-03-13

**Authors:** Kun Li, Zhiting Chen, Yanwen Qin, Yong-Xiang Wei

**Affiliations:** Department of Otolaryngology, Beijing AnZhen Hospital, Capital Medical University, Beijing 100029, China

## Abstract

**Background:**

Obstructive sleep apnea (OSA) is a common disease. It can cause many serious complications. OSA may increase the risk of hypertension. However, the exact mechanism of OSA causing hypertension is not fully understood. YKL-40/chitinase-3-like protein-1 plays an important role in vascular injury, repair, and generation. We sought to explore the role of YKL-40 in endothelial dysfunction and hypertension in OSA patients.

**Methods:**

All subjects were examined by polysomnography (PSG) and the expression of YKL-40 in the plasm of the subjects was measured by luminex. Carotid intima-media thickness (CIMT) was measured by B-mode ultrasound.

**Results:**

According to the conditions of OSA and hypertension, we studied four groups of 157 subjects, including OSA group (OSA, N=77), OSA with hypertension group (OSA+HT, N=37), hypertension group (HT, N=20), and healthly group (Con, N=23). YKL-40 levels were significantly elevated in OSA, OSA+HT, and HT group compared to Con groups. We used the ROC to predict the sensitivity and specificity of YKL-40 in all OSA patients or all hyperpietic patients. For OSA patients, the AUC of YKL-40 is 0.807 (95% confidence interval 0.725–0.888;* p*<0.01). For hyperpietic patients, the AUC of YKL-40 is 0.656 (95% confidence interval 0.570–0.742,* p*=0.01). There was a significant correlation between the parameter of OSA and hypertension and YKL-40 (*P*<0.05) and a significant correlation between Max-CIMT and YKL-40 (*P*<0.05).

**Conclusion:**

Elevated circulating levels of YKL-40 are associated with hypertension in OSA patients. The specificity of YKL-40 suggests that it could be a potential biomarker for OSA and hypertension.

## 1. Introduction

Obstructive sleep apnea (OSA) is a common problem that affects adults [1]. The main danger of OSA is that it is closely related to the deterioration of cardiovascular diseases [2]. According to the data provided by the National Sleep Disorder Research Center, OSA may cause serious cardiovascular disease; nearly 400000 OSA patients die of cardiovascular disease every year, and OSA and its complications cause great personal and public health effects. Obstructive sleep apnea syndrome caused by cardiovascular disease and its prevention has become a widespread concern throughout the world [3]. The accurate biomarkers of risk early warning and treatment evaluation are becoming more and more important for patients with OSA, especially in patients with OSA who have been suffering from cardiovascular complications. OSA could induce chronic intermittent hypopnoea, leading to inflammation and oxidative stress [4]. Inflammation plays a crucial role in the process of OSA causing many complications. The search and validation of an accurate inflammatory biomarker is becoming more and more urgent for OSA patients and is particularly beneficial for the assessment of primary cardiovascular complications and the risk of OSA [5].

YKL-40 is secreted and expressed in macrophages, neutrophils, and fibroblasts like synovial cells and chondrocytes and is in a relatively stable level of expression in the healthy population of different races. The significant elevation of YKL-40 levels is closely related to the pathological changes of cardiovascular diseases and OSA [6]. In addition, increased carotid intima-media thickness in patients with cardiovascular disease or OSA is associated with the level of YKL-40 [7]. YKL-40 is not only important in inflammation but also plays a role in vascular endothelial dysfunction and atherosclerosis. Increased YKL-40 levels affect cell migration and tissue remodeling, resulting in vascular endothelial dysfunction and injury [8, 9]. The specific causes of OSA and hypertension have not yet been fully elucidated. But many people have suggested that accurate biomarkers can be used as indicators for prevention and treatment of patients, which can effectively prevent the formation and development of OSA and hypertension.

YKL 40 plays an important role in vascular injury, repair, and generation. The exact mechanism of OSA causing hypertension is not fully understood. In addition, our research's purpose is highlighting the relationship between YKL-40 and hypertension in OSA patients.

## 2. Methods

### 2.1. Subjects and Blood Sample

We screened 116 participants including normal controls (Con, n=23), OSA alone (OSA, n=77), OSA patients with hypertension (OSA+HT, n=37), and patients with hypertension but without OSA (HT, n=20). All participants with a history of malignant tumour, diabetes mellitus, and chronic renal diseases were excluded. Our study was approved by the ethics committee of the hospital (Project identification code: 2017005) and was conducted in line with the Declaration of Helsinki. Informed consent was obtained for experimentation with human subjects. Venous blood sample was obtained before 9 am. Plasm was separated with centrifugation and stored for further analysis.

### 2.2. Measurement of PSG

OSA patients have upper airway obstruction, or partial obstruction, that affects sleep structure, leading to sleep fragmentation, daytime sleepiness, and repeated episodes of chronic intermittent hypoxia. Polysomnography (PSG) was used to measure the severity of OSA and hypoxaemia. The diagnostic standard of OSA is defined by the apnoea hypopnoea index (AHI) as > 5 events/hour, but this standard is further subdivided into mild (5 < AHI ≤15 events/hour), moderate (15 < AHI ≤ 30 events/hour), and severe (AHI > 30 events/hour). The diagnostic standard for hypoxaemia is defined as the lowest oxygen saturation (LOS), but this standard is further subdivided into mild (0.85≤ LOS < 0.9), moderate (0.65 ≤ LOS < 0.85), and severe (LOS<0.65).

### 2.3. Carotid Artery Ultrasound

Measurement of the carotid intima-media thickness (CIMT) was performed on all subjects using a colour Doppler diagnostic instrument (Toshiba Aplio 500 ultrasound scanner). The CIMT was defined as the distance between the blood-intimae and the media-adventitia interface on the wall of the artery. The study protocol and criteria were based on current sonographic guidelines. A max-CIMT below 0.9 mm was normal, a max-CIMT greater than 0.9 mm was considered to be intimal thickening, and a max-CIMT greater than 1.3 mm was considered atherosclerotic plaque [10, 11]. Each scan was made by the same investigator who was blinded to the patients' clinical data.

### 2.4. YKL-40 Assay

The plasm molecules were quantitated by using Human Magnetic Luminex Screening Assay (R&D Systems, Minneapolis, MN, USA). The concentrations of YKL-40 in plasm were determined according to the manufacturer's instructions. Acquisition was performed on the Bio-Plex system (Bio-Rad Laboratories) with the Bio-Plex 3D reader (Luminex FlexMAP 3D) in combination with xPONENT software version 4.2 (Luminex).

### 2.5. Statistical Analysis

Data are presented as the mean±SEM or as raw numbers, and the normality of variables was evaluated using the Shapiro Wilk test. For normally distributed data, we used one-way ANOVA and the least significant differences (LSD) post hoc multiple comparisons test. We used the ROC to analyse the diagnostic ability of plasm YKL-40 levels in all OSA patients or in all hyperpietic patients. The correlations among YKL-40 levels and AHI, LOS, DBP, SBP, lipid parameters, and max-CIMT were analysed using Spearman's correlations. Our data were processed with SPSS 13.0 (SPSS, Inc.). Statistical significance was defined as* P* < 0.05.

## 3. Results

### 3.1. Subjects Characteristics

Total cholesterol (TC), triglycerides (TG), and low-density lipoprotein cholesterol (LDL-c) levels in the OSA, OSA+HT, and HT groups were higher than in the Con group. The levels of C-reactive protein (CRP) and Hcy in the HT, OSA+HT, and OSA groups were not significantly higher than in the Con group. In addition, no difference in max-CIMT was found between the OSA and HT group (Table 1).

### 3.2. YKL-40

**Figure 1:**
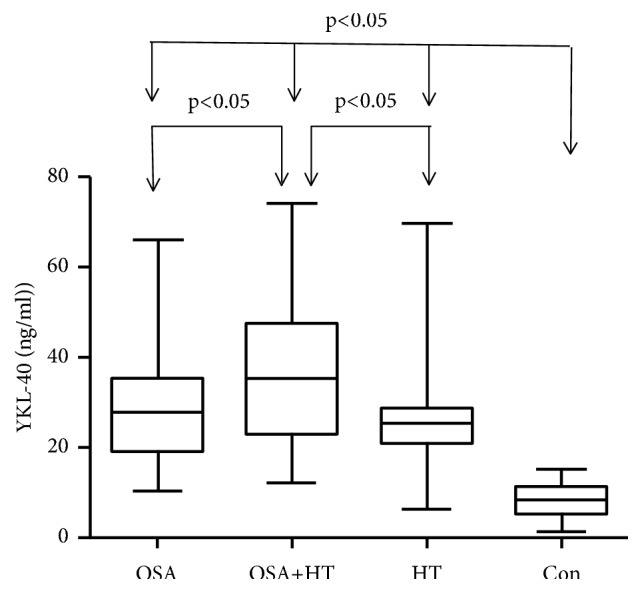
*The relative expression levels of YKL-40 in the Con, OSA, OSA+HT, and HT groups.* The horizontal lines indicate the mean. P<0.05 was considered significant. Plasma levels of YKL-40 in OSA+HT group were significantly higher than the other groups. OSA: obstructive sleep apnea patient; HT: hyperpietic.

Plasma YKL-40 was significantly elevated in OSA, OSA+HT, and HT groups compared to Con group; moreover, plasma YKL-40 was significantly elevated in OSA+HT group compared to OSA and HT groups. However, we found no significance between OSA groups and HT group (Figure 1).

### 3.3. Correlations between YKL-40 and Clinical Parameters

Correlations between plasm YKL-40 and clinical parameters were analysed using the Spearman's correlations. The analysis showed that the levels of plasma YKL-40 were correlated with TG and TC. SBP and DBP were correlated with the level of plasma YKL-40. The expression of plasm YKL-40 appeared to be critical to hypertension in our study, YKL-40 was not correlated with max-CIMT, and AHI and LOS were correlated with max-CIMT (Tables 2 and 3).

### 3.4. ROC Analysis of the Sensitivity and Specificity of YKL-40 in OSA or Hyperpietic Patients

**Figure 2:**
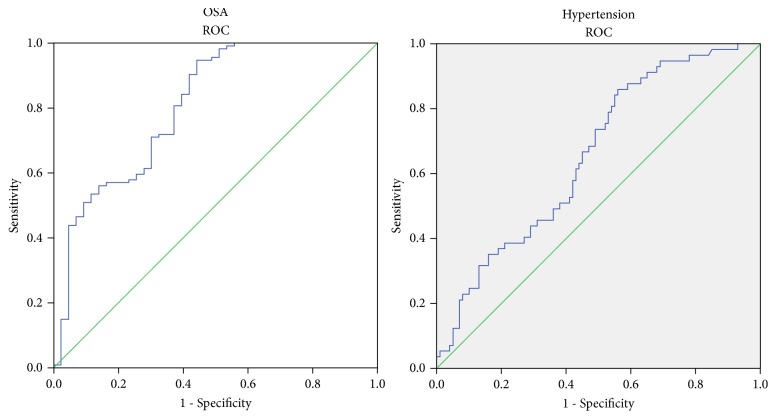
ROC curves for the sensitivity and specificity of YKL-40 associated with OSA and hypertension.

We used the ROC to analyse the sensitivity and specificity of YKL-40 in all OSA patients or all hyperpietic patients. For OSA patients, including the OSA and OSA+HT groups from all subjects, the AUC of YKL-40 is 0.807 (95% confidence interval 0.725–0.888; p<0.01). For hyperpietic patients, including the OSA+HT and HT subjects, the AUC of YKL-40 is 0.656 (95% confidence interval 0.570–0.742, p=0.01) (Figure 2).

## 4. Discussion

In our study, we found that the YKL-40 expression level in OSA patients with hypertension was significantly higher than that in patients with normal blood pressure and OSA. Moreover, the YKL-40 expression level of OSA patients with hypertension, OSA patients, and hypertension patients was significantly higher than that of the normal group. ROC analysis confirmed that the expression level of YKL-40 has a unique expression pattern in OSA patients and hypertensive patients. Correlation analysis showed that the YKL-40 expression level was significantly correlated with SBP and DBP and was also significantly correlated with AHI and LOS.

The characteristics of OSA have obvious negative effects on the pathogenesis of hypertension: intermittent hypoxia, oxidative stress, inflammation, and endothelial dysfunction, among others [12]. Marco M. Ciccone's study included 32 nonobese subjects, including 16 with moderate to mild OSA and 16 with snoring but without OSA and tested endothelial function and carotid intima-media thickness in all subjects. Their final findings suggest that OSA may affect the progression of atherosclerosis. Diagnosis, treatment, and prevention of OSA are very important, which can eliminate early the serious complications of OSA [13].

Previous studies have confirmed the relationship between arterial stiffness and left ventricular structure and function in patients with hypertension. Hypertension may cause left ventricular diastole and lead to heart disease. Early Doppler echocardiography is the best tool for diagnosing left ventricular diastolic dysfunction, which can provide a noninvasive warning for patients with hypertension [14]. So far, the exact mechanism of OSA induced hypertension is not fully understood. YKL-40 plays an important role in vascular injury, repair, and generation. We sought to explore the role of YKL-40 in endothelial dysfunction and hypertension in OSA patients. If YKL-40 expression level can predict the future risk of hypertension in OSA patients, then it can provide a reliable early warning index for the prevention of hypertension in OSA patient. Authentication and verification of circulating biomarkers is critical and especially beneficial in primary and medium hypertension and OSA risk assessment [15]. The significant elevation of YKL-40 levels is closely related to the pathological changes of cardiovascular diseases and OSA [16, 17]. In addition, increased carotid intima-media thickness in patients with cardiovascular disease or OSA is associated with the level of YKL-40 [18]. In recent years, Onofrio Resta and his colleagues have evaluated the consequences of the relationship of OSA and hypertension on carotid intima-media thickness and on inflammatory markers of atherosclerosis in four groups of subjects (controls; OSA patients without hypertension; hypertension patients without OSA; patients with OSA and hypertension). Additionally, OSA and hypertension have an additive role in the progression of carotid atherosclerosis of inflammatory markers for atherosclerosis. Our purpose is the same as previous articles, and we also used the same group of subjects to evaluate another protein with early warning function: YKL-40. We provide a liable early warning index for the prevention of hypertension in OSA patients [19].

Our results demonstrated that max-CIMT had no relationship with the level of YKL-40, but correlation analysis showed that the expression level of YKL-40 was significantly correlated with SBP and DBP and was also significantly correlated with AHI and LOS. So the expression level of YKL-40 is significant in OSA and hypertensive patients and can be used as a biomarker for two diseases. Moreover, in OSA patients with hypertension, the expression level of YKL-40 is higher than that of patients with OSA and hypertension.

YKL-40 is a very significant inflammatory marker in acute and chronic inflammation, which is secreted by macrophages, neutrophils, and vascular smooth muscle cells (VSMC). Many researchers have demonstrated that YKL-40 levels are elevated in many inflammatory diseases, for instance, rhinitis, asthma, tumors, myelofibrosis, and chronic obstructive pulmonary disease (COPD). Hinks and his colleagues revealed that IL-5 production emphasized the function of YKL-40 in severe asthma. Research by Mads' group revealed that enhanced levels of YKL-40 may be associated with pathogenesis in myelofibrosis. Park showed that the expression of YKL-40 was upregulated in the surface epithelium and vascular endothelial cells of mild and moderate severe allergic rhinitis patients and its expression can be regulated by different cytokines. These cytokines have an important influence on nasal mucosa remodeling in allergic rhinitis. YKL-40 induces the inflammatory response and thus influences the progression and outcome of tumor growth [20–23]. In our result, we had found no significant correlation between CRP or Hcy and YKL-40 (*P*>0.05).

YKL-40 is not only important in inflammation but also plays a role in vascular endothelial dysfunction and atherosclerosis. Increased YKL-40 levels affect cell migration and tissue remodeling, resulting in vascular endothelial dysfunction and injury [8, 9]. Psoriasis is a chronic inflammatory disease and has a close relationship with endothelial dysfunction and atherosclerosis. The association between YKL-40 levels and endothelial dysfunction in psoriasis patients is supported by recent studies. Gamze and his colleagues suggested that the increase of YKL-40 can predict the incidence of cardiovascular disease in psoriatic patients. The study showed that YKL-40 level was significantly higher in patients with psoriasis and endothelial dysfunction than in healthy control group. Elevated levels of YKL-40 in patients with psoriasis are not only characterized by diseases which are related to inflammation, but also with endothelial function and cardiovascular disease, which are closely related. Hence, YKL-40 could be used as a biomarker for psoriatic patients [24]. Endothelial dysfunction plays a significant role in nephrotic syndrome. In Kocyigit's experiment, 69 patients with nephrotic syndrome and 20 normal subjects were enrolled. Endothelial dysfunction and arterial stiffness were assessed by the flow mediated dilatation and the pulse wave velocity, and YKL-40 levels were measured in all participants. YKL-40 level in patients with nephrotic syndrome was significantly higher than normal subjects. The relationship between nephrotic syndrome itself and YKL-40 protein is also very close. YKL-40 can be used as a predictor of urinary protein levels in patients with nephrotic syndrome [25]. In our results, we had found significant correlation between max-CIMT and YKL-40 (*P*<0.05).

In 1992, Stoohs and his colleagues found OSA-related changes can lead to atherosclerosis or coronary heart disease [26]. Meanwhile, clinical data and epidemiological studies have pointed out a close relationship between OSA and the occurrence and development of cardiovascular disease. Shahar examined 6424 participants who underwent polysomnography (PSG). The incidence of cardiovascular events in participants with OSA was significantly higher than in those without OSA, and the incidence of cardiovascular events is significantly increased with increased severity of OSA [5]. Nevertheless, current studies could not explain the mechanisms of how OSA causes the occurrence and development of cardiovascular disease. Hence, it is very important to find a method to predict the probability of occurrence of cardiovascular disease. Matsuura et al. [5] pointed out that the YKL-40 protein may play an important role in the occurrence and development of OSA through the complex interaction of cytokines and transcription factors. The experiment included 159 OSA patients and 104 normal controls. The severity of OSA was assessed with AHI, and YKL-40 protein levels were measured at the same time. The YKL-40 level was correlated with the severity of OSA disease. A total of 246 patients with OSA were included in Sui's study, including 234 patients with OSA complicated with cardiovascular disease and 112 patients with OSA not complicated with cardiovascular disease. Participants were examined by coronary angiography and coronary atherosclerosis index (CAI) assessment, and the YKL-40 level was measured by ELISA. The experimental results showed that the level of YKL-40 was closely related to the severity of OSA. Xin Wang discovered that the level of serum YKL-40 and Apnea Hypopnea Index have a significant association. They showed that YKL-40 may be an early sign of the severity of OSA. Epidemiologic analysis showed that changes in the level of YKL-40 protein can be used as an important predictor of OSA [27, 28]. Jafari and his colleagues showed that elevated YKL–40 levels in OSA patients were linked with endothelial dysfunction. Intima–media thickness (IMT) was a key indicator for evaluation of atherosclerosis, and ultrasound could measure arterial intima-media thickness and evaluate the severity of atherosclerosis. The severity of OSA is closely related to the level of YKL-40 expression, and the severity of OSA also has a very important relationship with atherosclerosis [29]. Akyuz A et al. [7] have noticed the associations between increased carotid intima-media thickness and AHI in patients with OSA. Akyuz's study included 50 patients with OSA and 38 normal subjects. IMT had a very important relationship with the severity of OSA. Endothelial dysfunction plays an important role in the development of atherosclerosis. Indeed, YKL-40 has an important role in endothelial dysfunction. In our results, we had found significant correlation between OSA and YKL-40 (*P*<0.05). AHI and LOS are a measure of the severity of OSA. YKL-40 might play an important role in the development of OSA.

OSA can lead to intermittent hypoxia, which can lead to oxidative stress and inflammation of the body; oxidative stress and inflammation can induce vascular endothelial dysfunction, which plays a key role in the development of atherosclerosis. Finally, this leads to the occurrence and development of cardiovascular events. YKL-40 are directly or indirectly involved in the above processes. The occurrence and severity of OSA is closely related to the expression of YKL-40. YKL-40 may be an important target toward understanding cardiovascular disease induced by OSA. In our results, we had found significant correlation between SBP/DBP and YKL-40 (*P*<0.05). YKL-40 might play an important role in the development of hypertension.

There were several limitations to our study. First, the sample size was relatively small, which might have compromised the accuracy of the results. Second, the majority of subjects were males; thus the results of this study cannot be generalized across genders. Third, neither intracellular nor extracellular experiments were performed to further validate the function of YKL-40.

## 5. Conclusion

Elevated circulating levels of YKL-40 are associated with hypertension in OSA patients. The specificity of YKL-40 suggests that it could be a potential biomarker for OSA and hypertension.

## Figures and Tables

**Table 1:** Patients' characteristics parameters. BMI: body mass index; SBP: systolic blood pressure, DBP: diastolic blood pressure; AHI: apnea hypopnea index; LOS: lowest oxygen saturation; *∗P* value significant between groups 1-3 and 4; Hcy: homocysteine.

	OSA	OSA+H	H	Con
	(N=77)	(N=37)	(N=20)	(N=23)
Age, year	44.18±12.18	48.16±10.38	52.50±17.20	43.78±14.35
Male, n	65	32	12	16
BMI, Kg/m^2^	26.82±3.78	27.86±3.24	24.53+3.70	23.24+3.43
SBP, mmHg	122.74±9.29	140.51±10.31	138.45±14.10	113.43±16.32
DBP, mmHg	79.95±8.14	90.03±7.10	85.65±9.42	71.13±13.74
AHI, event/h	39.87±25.66	47.69±26.90	3.06±1.32	2.47±1.27
LOS, %	76.94±13.90	75.32±12.25	91.45±2.68	92.36±1.86
TC, mmol/l	5.24±1.41	5.36±1.04	4.89±1.15	4.52±1.10
TG, mmol/l	1.71±1.03	2.41±1.77	2.17±2.29	1.00±0.56
HDL, mmol/l	1.50±1.24	1.25±0.54	1.19±0.25	1.49±0.71
LDL, mmol/l	3.26±0.95	3.29±0.97	2.81±0.85	2.71±1.21
Hcy, mmol/l	13.06±6.94	14.27±5.62	12.68±6.09	11.18±5.81
CRP, mmol/l	3.21±2.39	4.27±5.28	1.80±1.52	2.52±4.41
Max-CIMT; mm	1.23±0.44	1.25±0.19	1.11±0.23	*∗* 0.96±0.15
YKL-40, ng/ml	29.34±12.99	35.60±14.78	23.60±13.08	8.51±3.45

**Table 2:** Correlations between YKL-40 and parameters of OSA and hypertension. *∗*: correlation is significant at the 0.05 level (2-tailed). *∗∗*: correlation is significant at the 0.01 level (2-tailed).

			SBP	DBP	YKL40	AHI	LOS	CIMT	BMI
Spearman's rho	SBP	correlation coefficient	1.000	.752^*∗∗*^	.276^*∗∗*^	.130	-.157^*∗*^	.116	.249^*∗∗*^
		Sig.(2-tailed)	.	.000	.000	.105	.049	.147	.002
	DBP	correlation coefficient	.752^*∗∗*^	1.000	.257^*∗∗*^	.264^*∗∗*^	-.236^*∗∗*^	.123^*∗∗*^	.314
		Sig.(2-tailed)	.000	.	.001	.001	.003	.126	.000
	YKL40	correlation coefficient	.276^*∗∗*^	.257^*∗∗*^	1.000	.385^*∗∗*^	-.383^*∗∗*^	.283^*∗∗*^	.264^*∗∗*^
		Sig.(2-tailed)	.000	.001	.	.000	.000	.000	.001
	AHI	correlation coefficient	.130	.264^*∗∗*^	.385^*∗∗*^	1.000	-.875^*∗∗*^	.358	.514^*∗∗*^
		Sig.(2-tailed)	.105	.001	.000	.	.000	.000	.000
	LOS	correlation coefficient	-.157^*∗*^	-.236^*∗∗*^	-.383^*∗∗*^	-.875^*∗∗*^	1.000	-.313^*∗*^	-.564^*∗∗*^
		Sig.(2-tailed)	.049	.003	.000	.000	.	.000	.000
	CIMT	correlation coefficient	.116	.123	.283^*∗∗*^	.358^*∗∗*^	-.313^*∗∗*^	1.000	.225
		Sig.(2-tailed)	.147	.126	.000	.000	.000	.	.005
	BMI	correlation coefficient	.249^*∗∗*^	.314^*∗∗*^	.264^*∗∗*^	.514^*∗∗*^	-.564^*∗∗*^	.225^*∗∗*^	1.000^*∗∗*^
		Sig.(2-tailed)	.002	.000	.001	.000	.000	.005	.

**Table 3:** Correlations between YKL-40 and clinical parameters. *∗*: correlation is significant at the 0.05 level (2-tailed). *∗∗*: correlation is significant at the 0.01 level (2-tailed). Hcy: homocysteine.

			SBP	DBP	YKL40	TC	TG	HDL	LDL	HCY	CRP
Spearman's rho	SBP	correlation coefficient	1.000	.752^*∗∗*^	.276^*∗∗*^	.108	.328^*∗∗*^	-.194	.043^*∗∗*^	.056^*∗∗*^	.080
		Sig.(2-tailed)	.	.000	.000	.180	.000	.015	.595	.487	.321
	DBP	correlation coefficient	.752^*∗∗*^	1.000	.257^*∗∗*^	.172^*∗*^	.323^*∗∗*^	-.175^*∗∗*^	.082	.141^*∗∗*^	.060^*∗*^
		Sig.(2-tailed)	.000	.	.001	.032	.000	.028	.309	.078	.456
	YKL40	correlation coefficient	.276^*∗∗*^	.257^*∗∗*^	1.000	.148	.293^*∗∗*^	-.114^*∗∗*^	.060^*∗∗*^	.166	.250
		Sig.(2-tailed)	.000	.001	.	.064	.000	.154	.454	.038	.002
	TC	correlation coefficient	.108	.172^*∗*^	.148	1.000	.465^*∗∗*^	.150	.781^*∗*^	-.081	.119
		Sig.(2-tailed)	.180	.032	.064	.	.000	.060	.000	.312	.139
	TG	correlation coefficient	.328^*∗∗*^	.323^*∗∗*^	.293^*∗∗*^	.465^*∗∗*^	1.000	-.395^*∗∗*^	.274^*∗∗*^	-.074^*∗∗*^	.135^*∗∗*^
		Sig.(2-tailed)	.000	.000	.000	.000	.	.000	.001	.354	.094
	HDL	correlation coefficient	-.194^*∗*^	-.175^*∗*^	-.114	.150	-.395^*∗∗*^	1.000^*∗*^	.052^*∗*^	-.037	-.001
		Sig.(2-tailed)	.015	.028	.154	.060	.000	.	.521	.645	.988
	LDL	correlation coefficient	.043	.082	.060	.781^*∗∗*^	.274^*∗∗*^	.052	1.000	-.052	.047^*∗∗*^
		Sig.(2-tailed)	.595	.309	.454	.000	.001	.521	.	.517	.557
	HCY	correlation coefficient	.056	.141	.166^*∗*^	-.081	-.074	-.037	-.052	1.000^*∗*^	-.064
		Sig.(2-tailed)	.487	.078	.038	.312	.354	.645	.517	.	.427
	CRP	correlation coefficient	.080	.060	.250^*∗∗*^	.119	.135	-.001	.047	-.064^*∗∗*^	1.000
		Sig.(2-tailed)	.321	.456	.002	.139	.094	.988	.557	.427	.

## Data Availability

The data used to support the findings of this study are available from the corresponding author upon request.
